# Oral administration of *Limosilactobacillus ingluviei* C37 inhibits *Campylobacter jejuni* colonization in chicks

**DOI:** 10.3389/fmicb.2024.1491039

**Published:** 2024-11-28

**Authors:** Aito Murakami, Ayako Watanabe-Yanai, Taketoshi Iwata, Fu Namai, Takashi Sato, Tadashi Fujii, Takumi Tochio, Sutisa Khempaka, Takeshi Shimosato

**Affiliations:** ^1^Graduate School of Medicine, Science and Technology, Shinshu University, Nagano, Japan; ^2^Division of Zoonosis Research, National Institute of Animal Health, National Agriculture and Food Research Organization, Tsukuba, Japan; ^3^Food and Feed Immunology Group, Laboratory of Animal Food Function, Graduate School of Agricultural Science, Tohoku University, Sendai, Japan; ^4^Livestock Immunology Unit, International Education and Research Center for Food and Agricultural Immunology (CFAI), Graduate School of Agricultural Science, Tohoku University, Sendai, Japan; ^5^Department of Biomolecular Innovation, Institute for Biomedical Sciences, Shinshu University, Kamiina, Japan; ^6^Department of Gastroenterology and Hepatology, Fujita Health University, Toyoake, Japan; ^7^Department of Medical Research on Prebiotics and Probiotics, Fujita Health University, Toyoake, Japan; ^8^BIOSIS Lab. Co., Ltd., Toyoake, Japan; ^9^School of Animal Technology and Innovation, Institute of Agricultural Technology, Suranaree University of Technology, Nakhon Ratchasima, Thailand; ^10^Institute for Aqua Regeneration, Shinshu University, Kamiina, Japan

**Keywords:** *Limosilactobacillus ingluviei* C37, *Campylobacter jejuni*, gut microbiota, chicken, probiotics

## Abstract

As the global population continues to grow, so too does the demand for poultry meat. However, the concurrent increase in the prevalence of drug-resistant bacteria has stimulated interest in the search for alternatives to antibiotics in poultry and livestock agriculture. One potential strategy is the use of probiotics. In this study, we showed that prophylactic oral administration of *Limosilactobacillus ingluviei* C37 (LIC37) reduced *Campylobacter jejuni* colonization of the cecum in cage-raised chicks, without causing significant changes in the overall diversity of gut bacteria. Further, the abundance of *Blautia*, another genus of probiotic bacteria, increased in the gastrointestinal tract following ingestion of LIC37 by chicks. These findings suggest that LIC37 could potentially be used as a novel probiotic agent against *C. jejuni* in livestock production.

## 1 Introduction

The increasing global demand for poultry meat has been attributed, in part, to the ongoing increase in the human population (Tabashsum et al., 2023). The poultry industry has grown considerably in recent decades, and the population of poultry, particularly chickens, has increased more than five-fold in the last 50 years (Mottet and Tempio, 2019). At the same time, poultry farms have adopted various techniques, such as genetic selection and the use of antibiotics, to maximize both productivity and quality while minimizing costs and production time (Siegel, 2014; Gadde et al., 2017; Carney et al., 2022).

In this context, a tight relationship has been observed between poultry growth efficiency, expressed as the feed conversion ratio (FCR), and the microbiota of the gastrointestinal tracts (GIT) of these animals (Stanley et al., 2012). Notably, it has been reported that the diversity of the GIT microbiome is inversely related to the FCR value (Diaz Carrasco et al., 2019). Antibiotics have been widely used as growth promoters in livestock agriculture (Gadde et al., 2017). However, excessive use of these agents has promoted the spread of antibiotic-resistant pathogenic bacteria, with concomitant concerns that these organisms may be transmitted to humans as zoonoses (García et al., 2020). As a result, the use of antibiotics as growth promoters has been restricted or even banned in many countries. Consequently, there has been increasing interest in exploring alternatives to the use of antibiotics in promoting livestock growth (Bahaddad et al., 2023).

Another issue in avian agriculture is infection by *Campylobacter jejuni*, a bacterium that is infamous for causing foodborne illness in humans. Infection by *C. jejuni* is primarily associated with the handling and consumption of raw or undercooked meat, and typically results in symptoms such as intense (and sometimes bloody) diarrhea, fever, and abdominal pain that can last from 7 to 10 days (Kemper and Hensel, 2023). According to the World Health Organization (WHO), the global burden of *Campylobacter* infection is 7.5 million cases per day (World Health Organization (WHO), 2013). A key strategy for reducing clinical cases of *C. jejuni* infection is to control and decrease the prevalence of this bacterium at the farm level. Studies have shown that an approximately 10-fold reduction in *C. jejuni* populations on chicken carcasses can significantly decrease the incidence of campylobacteriosis in humans (Rosenquist et al., 2003; Iwata et al., 2023).

Probiotics, defined as “live microorganisms which, when administered in adequate amounts, confer a health benefit on the host” (Hill et al., 2014), constitute an attractive alternative to antibiotics and a valid approach to decreasing the occurrence of *C. jejuni*. Some probiotics have been shown to improve feed intake and digestion while stimulating the immune system, promoting growth in livestock, including poultry (Krysiak et al., 2021). In addition, several probiotics are known to be beneficial in countering *C. jejuni* (Arsi et al., 2015; Mohan, 2015; Nishiyama et al., 2015). In Arsi et al. (2015), several bacterial strains were isolated from the ceca of birds and tested in an *in vivo* challenge in poultry against *C. jejuni*. They demonstrated a reduction of approximately 1- to 2-log units in *C. jejuni* abundance following oral administration of *Bacillus* spp. and *Ligilactobacillus salivarius* subsp. *salivarius* and *L. s. salicinius*.

The probiotic bacterium *Limosilactobacillus ingluviei* (formerly *Lactobacillus ingluviei*), a heterofermentative member of the lactic acid bacteria, was first isolated from the crop of a pigeon (Baele et al., 2003; Zheng et al., 2020). Previous studies have investigated *L. ingluviei* for its potential anti-*Salmonella* activity (Thomas et al., 2019) and growth-promoting effects (Angelakis and Raoult, 2010; Angelakis et al., 2012). The C37 strain of *L. ingluviei* (LIC37) was independently isolated in our laboratory from the intestines of broiler chickens, and we previously have reported the *in vitro* characteristics of this isolate, including its growth characteristics and potential utility as a probiotic agent (Tsukagoshi et al., 2020; Sirisopapong et al., 2023). However, the *in vivo* activity of LIC37 and its effects on host health have not yet been clarified.

In the present study, we sought to evaluate the potential application of LIC37 as a probiotic agent in chicks. Specifically, we observed the inhibitory impacts of this isolate on colonization of the chick GIT by *C. jejuni*, and clarified the associated changes in the cecal microbiota of chicks administered LIC37. We demonstrated that *C. jejuni* infection in these animals was strongly suppressed and that primary effect of LIC37 administration on the GIT microflora was an increase in the cecal abundance of LIC37, with no other significant changes in the bacterial diversity in this organ. In addition, we observed that LIC37 administration may induce the growth of a beneficial bacterium belonging to the genus *Blautia*. The results suggested that LIC37 may have potential as a probiotic agent protecting against *C. jejuni* on poultry farms.

## 2 Materials and methods

### 2.1 Isolation of *L. ingluviei*

Bacterial samples were isolated from fresh ileum and cecum, and the resulting samples were identified to species by 16S rRNA sequencing, as reported previously (Tsukagoshi et al., 2020). Briefly, samples obtained from healthy broiler chickens were diluted ten-fold in phosphate-buffered saline (PBS). Aliquots (100 μL) were spread on de Man, Rogosa, and Sharpe (MRS) agar [Becton and Dickinson and Company (BD), Franklin Lakes, NJ] under anaerobic conditions using AnaeroPacks (Mitsubishi Gas Chemical Company, Inc., Tokyo, Japan), and the inoculated plates were incubated anaerobically for 48 h at 37°C. The resulting colonies of Gram-positive rod-shaped, catalase-negative bacteria were identified and purified, and the isolates then were the stored at −80°C in medium containing 20% glycerol. After extracting DNA and amplifying the 16S rRNA gene, the DNA was denatured. The 16S rRNA gene amplicons were sequenced using a genetic analyzer. The obtained sequences were then compared with published bacterial sequences using the BLAST tool.^1^

### 2.2 Culture of LIC37

LIC37 was pre-cultured overnight under anaerobic conditions at 37°C in liquid MRS medium, and then subcultured in liquid MRS medium for another 24 h under the same conditions. The resulting cells were pelleted by centrifugation (8,000 × *g*, 5 min, 4°C), washed twice with sterile water, and then resuspended in PBS.

### 2.3 Preparation of *Campylobacter jejuni*

*Campylobacter jejuni* Strain 11–164 was isolated from chickens reared in Japan and cultured in Mueller-Hinton broth (MHB) at 42°C under microaerophilic conditions. Specifically, the strain was pre-cultured for 48 h, then subcultured in MHB for another 18 h. The resulting cells were pelleted by centrifugation (5,000 x *g*, 5 min, room temperature), washed once with sterile PBS, and then resuspended in PBS.

### 2.4 Management of experimental chicks

Newly hatched 1-day-old specific pathogen-free (SPF) White Leghorn chicks were obtained from Nisseiken Co., Ltd. (Tokyo, Japan). In our experiment, animals were housed three to a cage under ambient temperature and light conditions, with lights on from 6:00 am to 11:00 pm. Throughout the experiment, the chicks were closely monitored twice daily to ensure their welfare. Feed and water were provided *ad libitum*.

### 2.5 Experimental design

**Figure 1:**
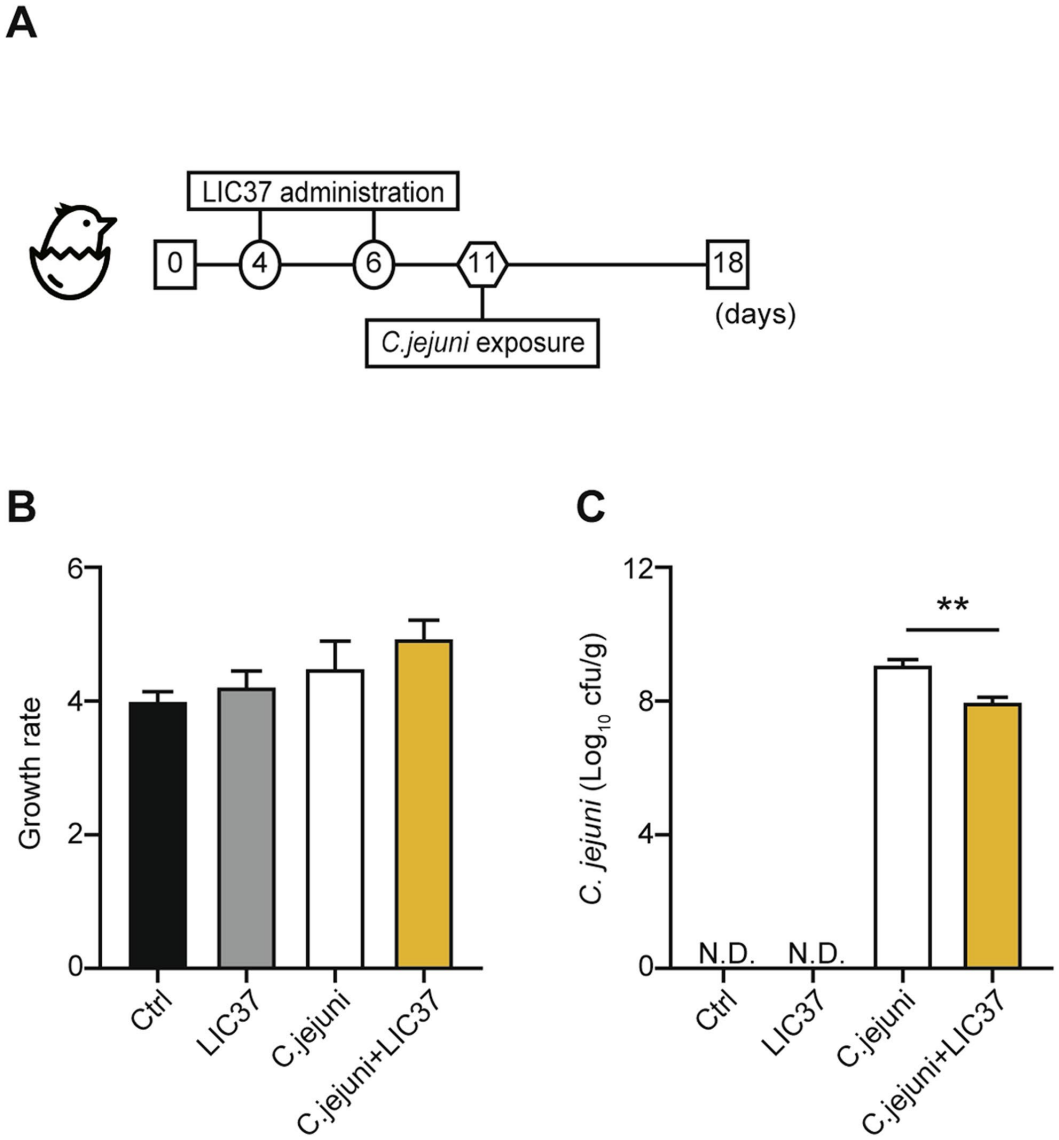
Body weight and abundance of colonizing *Campylobacter jejuni*. (A) Schematic diagram of the *in vivo* study. Chicks were divided into 4 experimental groups (Ctrl; *N* = 3, *Limosilactobacillus ingluviei* C37 (LIC37); *N* = 4, *C. jejuni*, and *C. jejuni* + LIC37; *N* = 6 each). The LIC37 and *C. jejuni* + LIC37 groups received two doses of *L. ingluviei* C37 (LIC37) on Days 4 and 6. The *C. jejuni* and *C. jejuni* + LIC37 groups were infected with *C. jejuni* on Day 11. Animals were maintained in the study until Day 18, when chicks were euthanized and the cecal contents were collected. (B) Body weights were recorded at the start and end of the study, and changes in body weight (compared to baseline) were calculated. (C) The abundance of *C. jejuni* in the cecal contents was quantified by colony counts. Data are presented as the mean ± SE (Ctrl; *N* = 3, LIC37; *N* = 4, *C. jejuni*, and *C. jejuni* + LIC37; *N* = 6 each). **p < 0.01, indicate significant differences as determined by a two-tailed one-way ANOVA with *post hoc* Tukey–Kramer multiple comparisons tests.

Two independent chick-infection experiments were conducted to assess the effects of LIC37 on the abundance of *C. jejuni* in the chick intestinal tract. The experimental design is shown in Figure 1A. Chicks were assigned to four groups: a control group (Ctrl; *N* = 3), an LIC37 group (LIC37; *N* = 4), a *C. jejuni* group (*C. jejuni*; *N* = 6), and an LIC37/*C. jejuni* group (*C. jejuni* + LIC37; *N* = 6). The first experiment included 12 chicks distributed equally among the four groups, while the second experiment consisted of only the experimental groups (1 from the LIC37 group, 3 from the *C. jejuni* group and 3 from the *C. jejuni* + LIC37 group, *n* = 7). At 4 and 6 days of age, chicks were orally administered 10^9^ colony-forming units (CFU) of LIC37 per chick, using a stomach tube. The number of lactobacilli needed to maintain colonization of the chicken intestinal tract was sustained for 2 weeks following the initial dose of LIC37 (Angelakis and Raoult, 2010). At 11 days of age, the chicks were orally challenged with *C. jejuni* Strain 11–164 at a dose of 10^7^ CFU/chick, administered through a stomach tube. The dosage was determined based on the findings of our previous paper (Iwata et al., 2016; Iwata et al., 2023). At 18 days of age, three chicks from each group were euthanized, and their cecal contents were collected. All chicks were weighed on days 1 and 18, and body weight gains were calculated for both infection experiments.

### 2.6 Sample collection

Qualitative and quantitative analyses for *C. jejuni* were conducted on the cecal samples from all chicks at 18 days of age. For the quantitative bacteriological assessments, cecal contents (0.2 g/chick) were subjected to a 10-fold serial dilution with physiological saline. The dilutions were then plated on Campylobacter Blood-free Selective Agar Base (Catalog No. CM0739B; Oxoid, Ltd., Basingstoke, UK) supplemented with Modified CCDA Selective Supplement (Catalog No. SR0155E; Oxoid, Ltd.), prepared according to the manufacturer’s instructions. After incubation in a microaerophilic atmosphere for 48 h at 42°C, the resulting colonies were counted. A mean value from the three chick samples was calculated to determine the cell density in CFU per gram of cecal content.

### 2.7 16S ribosomal DNA V3–V4 sequencing and microbiota analysis

Bacterial DNA was isolated from all cecal samples using a NucleoSpin® DNA Stool kit (Macherey-Nagel, Duren, Germany) according to the manufacturer’s instructions. The 16S V3–V4 region of the rDNA genes was amplified from the total DNA of the cecal content as described previously (Okajima et al., 2021). Library construction was conducted as reported previously (Murakami et al., 2023). The resulting rDNA library was sequenced using an Illumina MiSeq system (Illumina Inc., San Diego, CA) according to the manufacturer’s instructions. Analysis of microbiota was then performed using the Quantitative Insights into Microbial Ecology version 2 (Qiime2) platform (Bolyen et al., 2019). Error correction and filtering to remove noisy, chimeric sequences, and singletons, were performed using the Divisive Amplicon Denoising Algorithm 2 (DADA2). The cleaned sequences were then clustered into amplicon sequence variants (ASVs) and taxonomic annotation was performed using a classifier based on the SILVA database [Release No. 138; (Bokulich et al., 2018)]. The *α*-diversity of the gut microbiota was assessed using Faith’s phylogenetic diversity (PD), the observed ASVs, and the Shannon diversity index. Additionally, principal coordinate analysis (PCoA) was determined to visualize patterns of microbial community structure. Difference in *β*-diversity were tested using the Weighted Unifrac index. The gut microbiota was further characterized by linear discriminant analysis (LDA) effect size (LEfSe) conducted on the Huttenhower Laboratory Galaxy server (version 2.0) using LDA scores.^2^

### 2.8 Statistical analysis

Where applicable, data are presented as the mean ± standard error (SE). Inferential statistical analysis was performed using Prism software (version 7; GraphPad Software, San Diego, CA). Statistically significant differences were estimated using a two-tailed ordinary one-way analysis of variance (ANOVA) followed by *post hoc* Tukey–Kramer multiple comparisons tests, as appropriate. *p* < 0.05 was considered statistically significant.

## 3 Results

### 3.1 Body weight and quantitation of *C. jejuni* colonization

The animal experiment, shown schematically in Figure 1A, consisted of 4 groups: the control (Ctrl) group (no dosing with LIC37, no challenge with *C. jejuni*), the LIC37 group (dosing with LIC37, no challenge with *C. jejuni*), the *C. jejuni* group (no dosing with LIC37, challenge with *C. jejuni*), and the *C. jejuni* + LIC37 group (dosed prophylactically with LIC37, challenged with *C. jejuni*). All groups showed a 3- to 5-fold increase in mean body weight from Days 1 to 18 (Figure 1B), and there were no significant differences in body weight gain among the groups. On Day 18, quantification of *C. jejuni* in the cecal content demonstrated a significantly lower abundance of the pathogen in the *C. jejuni* + LIC37 group (7.99 Log_10_ cfu/g) compared to the *C. jejuni* group (9.17 Log_10_ cfu/g) (*p* = 0.0029) (Figure 1C).

### 3.2 Dynamics of the cecum microbiota in response to LIC37 administration

**Figure 2:**
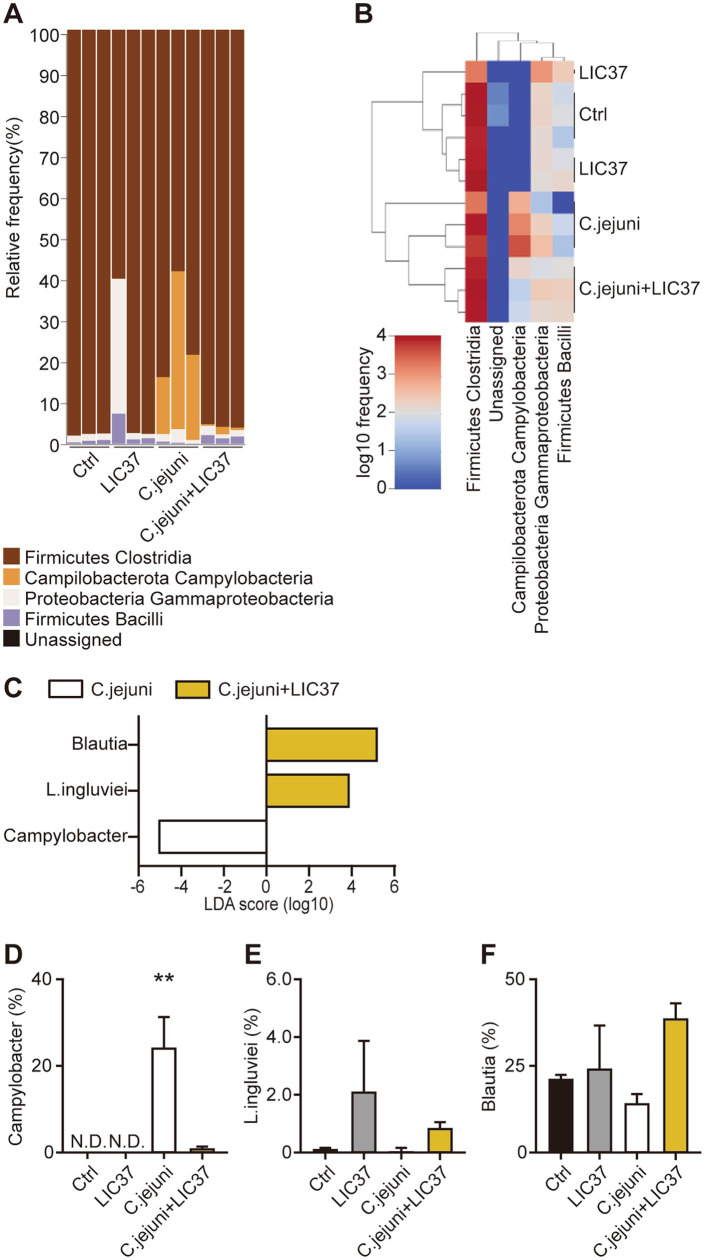
Dynamics of the cecal microbiota following *Limosilactobacillus ingluviei* C37 (LIC37) administration. (A) The bacterial composition (%) of the cecal contents at the class level is shown as a bar plot. (B) The heatmap shows significant changes in the cecal microbiota at the class level. Colors on the heatmap indicate the relative abundance of the various components of the cecal microbiota; red indicates increased abundance and blue indicates decreased abundance compared to the other two groups. (C) The linear discriminant analysis (LDA) effect size (LEfSe) method was used to identify taxa with the largest differences in abundance between the *C. jejuni* and *C. jejuni* + LIC37 groups. Bacteria with increased abundance in the indicated group are shown in the horizontal bar chart. (D–F) The proportions (%) of *C. jejuni*, *L. ingluviei,* and *Blautia* in the cecal contents in each group are shown. Data are presented as the mean ± SE (*N* = 3). **p < 0.01, indicate significant differences as determined by a two-tailed one-way ANOVA with *post hoc* Tukey–Kramer multiple comparisons tests. nd, no data.

The results of the taxonomic analysis are presented as a bar plot generated using QIIME2 (Figure 2A). The figure shows the bacterial composition at the class level in the cecal contents of each animal from each group. As expected, the *Campylobacter* bacteria were absent from the cecal contents of the Ctrl and LIC37 groups, but comprised up to 40% of the cecal microbiota in the *C. jejuni* group. Prophylactic dosing with LIC37 effectively eliminated *C. jejuni* colonization in the cecal contents of the *C. jejuni* + LIC37 group. Significant changes in the cecal microbiota at the class level were observed using a heatmap, confirming the depletion of *C. jejuni* in the cecal contents of the *C. jejuni* + LIC37 group compared to the *C. jejuni* group (Figure 2B). In addition, changes in the dynamics of the gut microbiota of chicks infected with *C. jejuni*, both with and without LIC37 administration, were analyzed using the linear discriminant analysis (LDA) effect size (LEfSe). Using the LDA score, we evaluated the dynamics of the gut microbiota at the genus and species levels and observed significant differences in three bacterial classes between the *C. jejuni* and *C. jejuni* + LIC37 groups (Figure 2C). As expected, the LDA score for LIC37 was elevated by approximately 4 logs, while that for *C. jejuni* was decreased by approximately 5 logs in the *C. jejuni* + LIC37 group compared to the *C. jejuni* group. Additionally, the LDA score for *Blautia* was increased by approximately 5 logs in the *C. jejuni* + LIC37 group compared to that in the *C. jejuni* group. Statistical analysis of the proportions (percentages) confirmed that the difference in *Campylobacter* abundance was statistically significant (Figures 2D–F).

### 3.3 Effect of oral administration of LIC37 or infection with *C. jejuni* on GIT bacterial diversity

**Figure 3:**
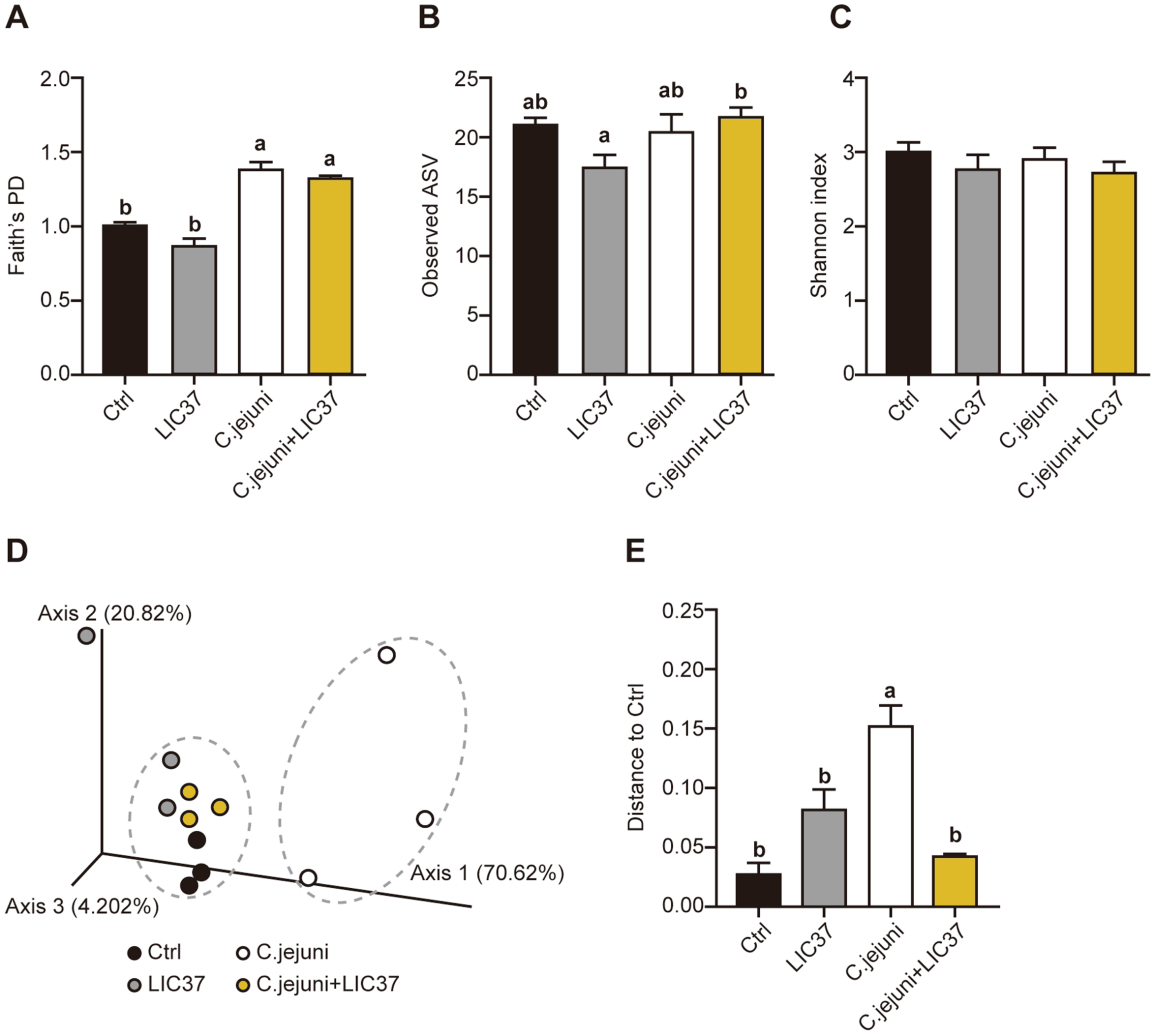
Effect of oral administration of *Limosilactobacillus ingluviei* C37 (LIC37) or infection with *Campylobacter jejuni* on gut microbiome diversity. Cecal contents were collected from chicks on Day 18 for rDNA sequencing and diversity analysis. The diversity of the cecal microbiota for each group were assessed using three measures of *α*-diversity: (A) Faith’s phylogenetic diversity (PD), (B) Amplicon sequence variants (ASVs), and (C) the Shannon index. (D) Principal coordinate analysis (PCoA) was performed based on the Weighted Unifrac distances at the ASV level. Dashed circles indicate clusters of similar points. (E) Significance testing of *β*-diversity differences was performed using the Weighted Unifrac index. Data are presented as the mean ± SE (*N* = 3). Significant differences are indicated by different symbols; statistical significance was determined using a two-tailed one-way ANOVA with *post hoc* Tukey–Kramer multiple comparisons tests.

Terminal cecal content samples were subjected to next-generation sequencing (NGS) and diversity analysis. The value of Faith’s phylogenetic diversity (PD) was significantly higher in both the *C. jejuni* group and *C. jejuni* + LIC37 group compared to the other groups; however, no significant differences in this parameter were observed between the *C. jejuni* and *C. jejuni* + LIC37 groups (Figure 3A). In the observed ASVs, significant differences were observed between the LIC37 group and the *C. jejuni* + LIC37 group (Figure 3B). Conversely, no significant differences in the Shannon diversity index (*α*-diversity) were observed among the four groups (Figure 3C). Next, we examined the Weighted Unifrac *β*-diversity results for all sample groups (Figures 3D,E). Based on the PCoA results, the gut microbiota of the *C. jejuni* group exhibited a distinct trend compared to the other groups (Figure 3D). Additionally, β-diversity was significantly higher in the *C. jejuni* group compared to the other groups (Figure 3E). Based on these findings, we inferred that LIC37 restored the diversity of the gut microbiota in *C. jejuni*-infected chicks.

## 4 Discussion

*L. ingluviei*, a novel probiotic lactic acid bacteria, has been shown to have beneficial effects on chickens, including anti-*Salmonella* activity (Thomas et al., 2019) and growth-promoting effects (Angelakis and Raoult, 2010; Angelakis et al., 2012). LIC37, a strain of *L. ingluviei* isolated from broiler chickens, has demonstrated immunological effects *in vitro* (Tsukagoshi et al., 2020; Sirisopapong et al., 2023). In this study, we examined the potential utility of LIC37 in maintaining livestock. Specifically, we investigated the *in vivo* effects of LIC37 administration on *C. jejuni* infection and the associated changes in the cecal microbiota of chickens.

We first tested the effects of LIC37 on growth promotion, a common criterion for assessing probiotics (Krysiak et al., 2021). In contrast to previous findings with other strains of *L. ingluviei* (Angelakis and Raoult, 2010; Angelakis et al., 2012), we observed that LIC37 administration did not significantly enhance chicken weight gain over the course of the study (Figure 1). However, no adverse effects, such as attenuation of weight gain, were detected in the present study. Moreover, there were no observed signs or symptoms of morbidity attributable to the administration of LIC37 alone. In fact, the growth rate in the LIC37 group was comparable to or slightly better than that in the control group. These findings not only underscore the potential for LIC37 as a probiotic, but they also suggest that this strain is safe for use.

*Campylobacter jejuni* is well known for its role in foodborne illnesses. This pathogen colonizes the GITs of both wild and domestic warm-blooded animals, with poultry acting as the primary reservoir (Skarp et al., 2016). Historically, antibiotics have been employed to control *C. jejuni* in the poultry industry, but there is growing concern about the overuse of antimicrobials, particularly given the increased prevalence of drug-resistant bacteria (Mingmongkolchai and Panbangred, 2018). Consequently, the poultry industry is striving to reduce or eliminate antibiotic use, both for therapeutic applications and as growth promotors, and is exploring alternative strategies for controlling *C. jejuni* in poultry production (Al Hakeem et al., 2022).

Several studies have investigated probiotics with the potential to reduce *C. jejuni* colonization and infection (Eeckhaut et al., 2016; Saint-Cyr et al., 2017; Smialek et al., 2018; Massacci et al., 2019). For example, Smialek et al. (2018) demonstrated that the supplementation of chicken feed with Lavipan, a probiotic consisting of several *Lactobacillus* strains, reduced *C. jejuni* invasion in the GIT of broiler chickens. In addition, the use of bacilli as probiotics in host species other than those from which the bacteria were originally derived has become increasingly common (Mingmongkolchai and Panbangred, 2018), despite evidence suggesting that strains with the same ecological origin may be more effective (Krysiak et al., 2021). Therefore, evaluating the safety of various bacilli for consumption by livestock remains crucial (Hong et al., 2005). In this study, we used LIC37, a strain that was originally isolated from broiler chickens (Tsukagoshi et al., 2020). We sought to increase the proportion of this bacterium in the gut of recipient chickens by oral administration alone, without any additional supplementation with any chemicals or bacteria derived from other host species. We propose that this strategy is likely the most effective for preserving the health of poultry, humans, and the surrounding environment. This strategy significantly reduced the number of *C. jejuni* not only in the cecum, but also in the ileum and the jejunum (data not shown). Furthermore, we evaluated the balance of the cecal microbiota in animals infected with *C. jejuni*, both with and without prophylactic administration of LIC37. Our findings showed that there were no significant changes in the composition of the cecal microbiota of infected animals, except for the proportion of *C. jejuni* (Figure 3). The observed changes did not affect the *α*-diversity of the cecal microbiota, which measures species diversity within a community (Figure 3C). However, the *β*-diversity, which assess the degree of diversity between groups, showed that the cecal microbiome in both the LIC37 and *C. jejuni* + LIC37 groups resembled that observed in the Ctrl group, while the *C. jejuni* group formed a distinct cluster (Figure 3). Our results indicate that increasing the abundance of LIC37, itself a chicken isolate, to approximately 1% of the cecal microbiota efficiently prevents *C. jejuni* infection.

Other studies have shown that individual strains of *Lactobacilli* are no longer detectable in the cecum of inoculated chicks at 1 week after a single oral inoculation at hatching (Kubasova et al., 2019). We therefore considered that the ability of LIC37 to attach to intestinal epithelial cells and persist in the GIT for extended periods should be investigated. Considering that LIC37 is a native component of the chicken gut microbiome, we expected that this bacterium would persist in the GIT; however, distinguishing between administered bacteria and native bacteria was initially considered to be challenging. Despite this, the cecal abundance of *L. ingluviei* remained elevated in chicks administered LIC37, even more than a week after oral administration. Analysis of the LDA scores revealed that three classes of bacteria showed significant differences in abundance between the cecal contents of the *C. jejuni* and *C. jejuni* + LIC37 groups, with *L. ingluviei* being one of the most abundant bacteria in the cecal content of the *C. jejuni* + LIC37 group (Figure 2).

Members of the *Limosilactobacilli*, including *L. ingluviei,* are known to be more abundant in the ileum compared to the cecum as the ileal microbiome has a higher prevalence of facultative anaerobes (Sengupta et al., 2013; Kubasova et al., 2021). We therefore expected that LIC37 would show excellent adhesion to the intestinal wall, including the cecum in chickens, thereby enhancing the potential of LIC37 as a novel probiotic against *C. jejuni* infection. Indeed, our *in vivo* results supported this expectation and corroborated our earlier *in vitro* analyses (Sirisopapong et al., 2023), which showed that LIC37 was capable of adhering to Caco-2 cells. Consequently, the present study clarified the potential application of LIC37 as an *in vivo* probiotic agent.

Separately, Saint-Cyr et al. (2017) demonstrated that oral treatment with *L. salivarius* SMXD51 every other day (Q2D) for 35 days exhibited anti-*C. jejuni* activity *in vivo*. In contrast, our study employed a simpler regimen of only two Q2D doses (on Days 4 and 6) to prevent *C. jejuni* colonization. However, our study did not follow the chicks to market age, when *C. jejuni* can reach densities of 1 × 10^9^ CFU per gram of cecal content, suggesting that the dosing schedule in our study was not optimized for long-term efficiency under field conditions (Al Hakeem et al., 2022). Therefore, further studies are necessary to evaluate the long-term impacts of LIC37 exposure on the gut microbiota in both chicks and adult chickens. Despite these limitations, previous studies have shown that a 2-log decrease in the number of *C. jejuni* on chicken carcasses can result in a 30-fold decrease in the incidence of *C. jejuni* infection in humans (Rosenquist et al., 2003; Hermans et al., 2011). Given that this pathogen typically colonizes animals at between 2 and 3 weeks of age (Pielsticker et al., 2012; Al Hakeem et al., 2022), we consider that our dosing regimen, involving LIC37 administration on Days 4 and 6, is likely to be beneficial and effective in preventing *C. jejuni* infection.

Although, *C. jejuni* colonization was mitigated by prophylactic dosing with LIC37, the underlying mechanism of this effect remains unknown. In general, there exist four possible (non-mutually exclusive) mechanisms whereby probiotics may prevent *C. jejuni* infection: (A) stimulation of cellular responses, (B) interactions between pathogens and pre-treated intestinal cells, (C) immunomodulatory effects, and (D) attenuation of intestinal inflammatory processes (Balta et al., 2022). In addition, bacteriocin activity has also been noted as a possible mechanism (Messaoudi et al., 2012; Zommiti et al., 2016). The findings of our previous study suggested that some component of LIC37 exhibited *in vitro* anti-inflammatory properties, which resulted in the suppression of the production of pro-inflammatory cytokines such as interleukin 6 and tumor necrosis factor *α* (Tsukagoshi et al., 2020). Therefore, we hypothesize that the *in vivo* efficacy of LIC37 in this study may involve mechanisms (B) and (C), although further analyses will be needed to confirm this.

On the other hand, we observed that only a limited number of bacterial species exhibited increased abundance in the LIC37-dosed group compared to the *C. jejuni* group, as assessed by LDA scores (Figure 2). Notably, the genus *Blautia*, which includes bacteria associated with metabolic and inflammatory diseases as well as biotransformation processes, accumulated in the cecal microbiota of the *C. jejuni* + LIC37 group (Liu et al., 2021). *Blautia* has shown potential as a probiotic; for example, mice administered *Blautia wexlerae* orally have demonstrated attenuation of both high-fat diet–induced obesity and diabetes, an effect that is linked to the unique amino-acid metabolism profile of this bacterium (Hosomi et al., 2022). Furthermore, it should be noted that members of the genus *Blautia* typically produce bacteriocins as secondary metabolites. Hatziioanou et al. (2017) showed that *Blautia obeum* A2-162 produces a novel antibiotic, nisin O, which exhibits activity against the enteric poultry pathogen *Clostridium perfringens* in the presence of trypsin (Sirisopapong et al., 2023). Additionally, oral administration of *Limosilactobacillus reuteri* and *L. fermentum*, which are related to *L. ingluviei*, has been reported to enhance the abundance of *Blautia* in the gut and fecal microbiota of humans, piglets, and mice (Burakova et al., 2022; Wang et al., 2022; Lin et al., 2023). In this study, the administration of LIC37 may have resulted in increases in the abundance of such beneficial bacteria, which might be advantageous for host health, but the mechanism of this effect remains unclear. Further studies will be needed to clarify the role of *Blautia* in the gut microbiota and to explore the relationship between the host microbiota and any bacterial pathogens, including *C. jejuni*.

In conclusion, the findings of the present study suggest that LIC37 may have utility as a probiotic in chicks, demonstrating preventive activity against *C. jejuni* infection. LIC37 demonstrated excellent adhesion to the cecal wall of chickens *in vivo,* persisting in the cecum for more than 1 week. Further, dosing with LIC37 led to an increase in the abundance of beneficial microorganisms, including *Blautia*. These findings indicate that that LIC37 could be a practical and effective probiotic agent against *C. jejuni* on poultry farms. However, the practical application of LIC37 as a probiotic will require further research to optimize the LIC37 exposure regimen, including the feeding method, timing, and dosage.

## Data Availability

The datasets presented in this study can be found in online repositories. The names of the repositories and accession numbers can be found at the DNA Data Bank of Japan (DDBJ; https://www.ddbj.nig.ac.jp/) under project PRJDB17376.

## Footnotes

^1^ http://www.ncbi.nlm.nih.gov/

^2^ https://huttenhower.sph.harvard.edu/galaxy
